# Estimation of parasitaemia in imported falciparum malaria using the results of a combined rapid diagnostic test. No big help from haematological parameters

**DOI:** 10.1186/s12936-023-04781-2

**Published:** 2023-11-16

**Authors:** Manuel Jesús Soriano-Pérez, Nerea Castillo-Fernández, Ana Belén Lozano-Serrano, María Pilar Luzón-García, José Vázquez-Villegas, María Isabel Cabeza-Barrera, Jaime Borrego-Jiménez, María José Giménez-López, Joaquín Salas-Coronas

**Affiliations:** 1 Tropical Medicine Unit. Hospital Universitario de Poniente, Ctra. de Almerimar, 31, 04700 El Ejido, Almería, Spain; 2 Hematology Unit. Hospital Universitario de Poniente, El Ejido, Almería, Spain; 3 Department of Nursing, Physiotherapy and Medicine. Faculty of Health Sciences, University of Almeria, Almeria, Spain

**Keywords:** Malaria, Traveller, Imported disease, Rapid diagnostic test, Migrants

## Abstract

**Background:**

Microscopy continues to be the mainstay for the evaluation of parasitaemia in malaria but requires laboratory support and microbiological experience. Other fast and simple methods are necessary.

**Methods:**

A retrospective observational study of imported malaria treated from July-2007 to December-2020 was carried out to evaluate the association between the degree of parasitaemia and both rapid diagnostic tests (RDT) reactivity patterns and haematological parameters. *Plasmodium falciparum* monoinfections diagnosed by peripheral blood smear and/or polymerase chain reaction (PCR),which also had a positive RDT result in the same blood sample, were included in the study.

**Results:**

A total of 273 patients were included. Most of them were male (n = 256; 93.8%) and visiting friends and relatives (VFR) travellers (n = 252; 92.3%). Patients with plasmodial lactate dehydrogenase (pLDH) or aldolase and histidine-rich protein 2 (HRP-2) co-reactivity (Pan/Pf pattern) had a parasitaemia range between 0 and 37% while those with just HRP-2 reactivity (*P. falciparum* pattern) had ranges between 0 and 1%. Not a single case of *P. falciparum* pattern was found for parasitaemia ranges greater than 1%, showing a negative predictive value of 100% for high parasitaemia. All the correlations between haematological parameters and parasitaemia resulted to be weak, with a maximum rho coefficient of -0.35 for lymphocytes and platelets, and of 0.40 for neutrophils-to-lymphocytes count ratio. Multivariate predictive models were constructed reflecting a poor predictive capacity.

**Conclusions:**

The reactivity pattern of RDT allows a rapid semi-quantitative assessment of *P. falciparum* parasitaemia in travellers with imported malaria, discriminating patients with lower parasite loads. Haematological parameters were not able to estimate parasitaemia with sufficient precision.

**Supplementary Information:**

The online version contains supplementary material available at 10.1186/s12936-023-04781-2.

## Background

Malaria is the most important parasitic disease in the world. Nearly half of the world's population lives in areas at risk for infection. In 2021, there were an estimated 247 million malaria new cases and about 619,000 related deaths [1]. *Plasmodium falciparum* is the species that most frequently produces severe symptoms and is responsible for most of the morbidity and mortality associated with the infection. Sub-Saharan Africa suffers the greatest burden of the disease, reporting 95% of all malaria cases and 96% of all malaria-related deaths globally occurred during the 2019–2020 period [1]. Nonetheless, as a result of migratory movements and international tourism, the problem has ceased to be exclusive to endemic areas. Indeed, cases of imported malaria are nowadays reported worldwide [2–4] and around 8,000 imported cases are reported annually in Europe [5].

Microscopic examination remains the “gold standard” for laboratory confirmation of malaria, not only because of its high sensitivity and specificity, but also because it allows identifying the species and quantifying the degree of parasitaemia. However, it requires skilled microscopists often not available in many health facilities. This is especially concerning outside endemic zones where most laboratorians may not be used to tropical diseases diagnostic tests requiring the use of microscopy visualization techniques. Molecular techniques, on the other hand, are usually available only in reference centres.

For all these reasons, rapid diagnostic tests (RDT) have become the preferred method for initial diagnosis since they are simple, fast, offer high sensitivity and specificity, are widely available, and do not require previous experience to interpret their results [6, 7]. Yet, they are not capable of measuring parasitaemia, one of the most important criteria for distinguishing severe malaria cases from non-serious ones. To overcome such problem, several researchers have tried to correlate the different patterns of RDT reactivity with the degree of parasitaemia, although the studies carried out so far are scarce [8, 9]. In a similar way, efforts have been made to correlate different haematological parameters with the severity of parasitaemia, obtaining very disparate and even contradicting results [10, 16]. Being continuous quantitative variables, haematological parameters could theoretically estimate parasitaemia much more accurately (if a high correlation with any of them could be found) than RDT, which offers qualitative results. Several studies in children have hypothesized that haematological ratios, parameters less used in routine clinical practice, could be more useful for this purpose than classic haematological variables [13].

The present study aims to evaluate the association between the degree of parasitaemia and both RDT reactivity patterns and haematological parameters in a cohort of patients with *P. falciparum* imported malaria.

## Methods

### Study design

A retrospective observational study of all malaria episodes treated at the Tropical Medicine Unit (TMU) of the Poniente Hospital (El Ejido, Almería, Spain) between July 2007 and December 2019 was conducted. All patients over 14 years of age with *P. falciparum* monoinfections diagnosed by peripheral blood smear and/or PCR, who also had a positive RDT result in the same blood sample, were included in the study.

### Setting of the study

The Poniente hospital serves an area located in Southern Spain that holds a population close to 300,000 people, with a migrant share of 21%. Migrants from sub-Saharan Africa, mainly from Western Africa, are a very large group within this foreign population, and malaria is one of the most frequently imported diseases diagnosed among them [4, 17]. Migrants were classified as VFR travellers or non-VFR migrants. Spain-based migrants travelling to their homeland to visit friends and relatives were considered VFR travellers, whereas those migrants travelling first time from malaria endemic areas to Europe where considered non-VFR migrants. Spain-born citizens living in an African country were considered expatriates.

Patients coming from endemic areas presenting with symptoms suggesting patent malaria were mostly diagnosed at the Emergency Department. In addition, malaria cases were also detected in the outpatient clinic for tropical diseases (TMU) as a result of the malaria screening tests that are offered to all migrants coming from endemic areas in the last 12 months, irrespective of the reason for consultation at the clinic.

### Parasitological analysis

Malaria diagnosis was made by means of RDT, direct microscopic examination of thin smear and / or molecular tests. In all cases, the tests were performed using peripheral venous blood samples extracted in 5 mL EDTA tubes.

Two different RDT were used along the study period: the BinaxNOW® malaria test and the SD Bioline Malaria Ag P.f/Pan®. The first one, used from 2007 to 2015, is an immunochromatographic assay that detects both histidine-rich protein 2 (HRP-2), an antigen specific to *P. falciparum*, and aldolase, a pan-malarial antigen found in all *Plasmodium* species. The second one is the SD Bioline Malaria Ag P.f/Pan® that detects both HRP-2 and pLDH (*Plasmodium* lactate dehydrogenase), a plasmodial enzyme expressed by all *Plasmodium* species, and was used in the second part of the study, from 2016 on. Reactivity for both antigenic bands was defined as the Pan/Pf pattern, while reactivity for just the HRP-2 band was defined as the Pf pattern. The degree or level of parasitaemia in the thin smear was expressed as the percentage of parasitized erythrocytes. Blood smear examinations were performed by several microscopist, all experienced in imported tropical pathology. For *Plasmodium* DNA detection, a conventional Nested Multiplex Malaria PCR (NM-PCR), capable of identifying four human malaria species (*Plasmodium vivax, Plasmodium falciparum, Plasmodium ovale* and *Plasmodium malariae*), was used [18]. Parasitaemia was considered 0% for all patients with a negative thin smear examination, but a positive molecular test.

### Haematological analysis

The haematological parameters were obtained from the same blood sample used for the malaria diagnostic test of each case. The values of haemoglobin (g/dL), leukocytes (/µL), lymphocytes (/µL), neutrophils (/µL), monocytes (/µL), eosinophils (/µL), and platelets (/µL) were recorded. Additionally, the ratios of monocytes-to- lymphocytes count (MLCR), and of neutrophils-to-lymphocytes count (NLCR) were calculated.

### Statistical analysis

A descriptive analysis of all variables was carried out. Quantitative variables were assessed through the mean, median and range. To describe qualitative variables, absolute and relative frequencies (%) were used. The normality of all the variables was checked by the Shapiro–Wilk or Anderson–Darling tests.

The predictive capability of parasitaemia to discriminate the rapid diagnostic test results was investigated through the ROC (receiver operating characteristic) curve and the Youden’s index. The best cut-off point was the one that maximized the value of the Youden’s index. In order to show the distribution of the RDT results regarding the level of parasitaemia, a first categorization of parasitaemia into four groups was made: < 0.01%, 0.01%-0.1%, > 0.1%-1%, and > 1%. These cutoff points were chosen to facilitate comparison with those other published studies that had used a similar categorization [8].

Due to the lack of normality in most of the variable distributions, the correlation between haematological variables and parasitaemia was assessed by means of the Spearman’s rank correlation coefficient (rho). To predict parasitaemia, a generalized linear model was proposed. In this work, it was assumed that the response variable followed a quasibinomial distribution (proportion data). In fact, the dependent variable was divided by 100, so its range was restricted to the closed interval 0 to 1. For all the cases, the covariates which were highly correlated (> 0**.**75) were deleted from the equation. The dataset was split into two subsets, the training set (75%) and the test or validation set (25%). The model was built applying tenfold cross validation (10-k CV) in the training set. This method requires to randomly divide the training dataset in 10 subsets. Each time, one subsample will be retained and used as a validation set, while the model will be set up using the rest of the subsamples. In 10-k CV, the cross-validation mechanism is repeated 10 times, and then, the 10 results are averaged to produce just one single estimation of the model. Afterwards, the model was verified in the test set. To assess the performance of the statistical learning process from the training data, the root mean square error (RMSE) was calculated in order to know how the predicted values fit the real data.

Finally, the level of parasitaemia was categorized into another four different groups: < 1%, 1%-2**.**5%, > 2**.**5%—4%, and > 4%. These categories follow the World Health Organization (WHO) severity criterion that considers parasitaemia > 2.5% as a mortality risk factor, and parasitaemia > 4% as hyperparasitaemia (6). The 1% limit was added to differentiate those patients with a very low parasite density among the rest of non-severe malaria cases (parasite density < 2.5%). The location parameters of all the haematological variables were compared between all these categories. Without the normality assumptions, Kruskal–Wallis rank sum test was used to analyse them, being followed by Nemenyi post-hoc test to establish between which two groups were the differences found. A predictive model was constructed to try to discriminate between parasitaemias < 1% and ≥ 1% following the same re-sampling strategy (tenfold CV). For this statistical model, selection of the variables was made following Akaike’s information criterion (AIC). The confusion matrix was obtained when the real observations were compared to the predicted ones in the validation dataset.

Statistical analysis have been carried out with R software (R Core Team 2020). The significance level was set up at 0.05.

## Results

### General characteristics

During the study period, 392 malaria episodes were treated. Infections by species other than *P. falciparum* and/or mixed infections were excluded from the analysis (n = 34). Those episodes in which the RDT was not performed with the same blood sample used for the blood smear and/or for the *Plasmodium* PCR test were also excluded (n = 85). Finally, 273 malaria episodes (69.6% of the total) met the study inclusion criteria.

The epidemiological, clinical and analytical characteristics of the patients included in the study are shown in Table 1. The majority were men (93.8%) with a mean age of 35 years. 268 patients (98.2%) were migrants of sub-Saharan origin, with 92.3% of them being VFR travellers. The most frequent countries of origin were Mali and Senegal (60.8% and 12.1%, respectively). For sub-Saharan patients, the mean stay in Spain was 117.35 months and the mean time since last visit to malaria endemic areas and the malaria episode was 2.96 months.

**Table 1:** General characteristic of patients included in the study

			Total of patients n = 273
Mean age in years (range;SD)			35 (14–71; 8.49)
Gender (number, %)			
Male			256 (93.8%)
Female			17 (6.2%)
Country of origin (number, %)			
Mali			166 (60.8%)
Senegal			33 (12.1%)
Ghana			18 (6.6%)
Equitorial Guinea			14 (5.1%)
Guinea Bissau			12 (4.4%)
Burkina Faso			8 (2.9%)
Gambia			6 (2.2%)
Guinea			6 (2.2%)
Spain			4 (1.5%)
Nigeria			3 (1.1%)
Cote d’Ivoire			1 (0.4%)
Cameroon			1 (0.4%)
Romania			1 (0.4%)
Type of traveller (number, %)			
VFR travellers			252 (92.3%)
Non-VFR migrants			16 (5.9%)
Expatriate			3 (1.1%)
Others			2 (0.7%)
Mean time living in Spain in months (range)			117.35 (1–360)
Time (months) to last visit to a malaria-endemic country (mean, range)			2.96 (1–156)
HIV infection			8 (2.9%)
Diagnosing services			
Emergency Department			258 (94.5%)
Tropical Medicine Unit			15 (5.5%)
ICU admission (number, %)			49 (17.9%)
Deaths (number, %)			0 (0.0%)
Laboratory test results (mean, range)			
Haemoglobin (g/dL)			13.3 (4.7 – 17.7)
Leukocytes (/microL)			5,719.5 (2,000 – 31,500)
Neutrophils (/microL)			3,880.5 (930 – 14,912)
Lymphocytes (/microL)			1,045 (158 – 14,096)
Monocytes (/microL)			462.1 (44–2,245)
Eosinophils (/microL)			80.4 (0–3,000)
Platelets (/microL)			111.9 (10–864)
Monocyte-Lymphocyte Ratio			0.5 (0.06–2.7)
Neutrophil–Lymphocyte Ratio			5.6 (0.1–29.9)
Parasitological test results			
Parasitaemia % (mean, range)			1.8 (0–37.4)
Smear microscopy (number,%)			
Positive			248 (90.8%)
Negative			25 (9.2%)
RDT (number,%)			
Pan + Pf			195 (71.4%)
Pf			78 (28.6%)

SD: standard deviation; VFR: visiting friends and relatives; ICU: intensive care unit; RDT: rapid diagnostic test

Most cases (94.5%) were diagnosed in the Emergency Department. The rest were diagnosed as a result of the implementation of the imported diseases screening protocol used in our Tropical Medicine Unit. Mean parasitaemia was 1.8% (range: 0%-37.4%). A 17.9% of the cases required admission to the Intensive Care Unit (ICU), most of them (80%) just because of hyperparasitaemia, with no other signs or symptoms of severe malaria. There were no deaths.

In 90.8% of patients (n = 248), diagnosis of malaria and of the *Plasmodium* species were confirmed by the peripheral blood smear results. For the 25 patients with a positive RDT but no detectable parasitaemia in the blood smear, confirmation was performed by PCR.

### Relation between RDT results and parasitaemia

Co-reactivity on the RDT for pLDH**/**aldolase and HRP-2 (Pan/Pf pattern) was observed in 195 patients (71.4%). The rest (28.6%; n = 78) showed reactivity just for HRP-2 (Pf pattern).

**Fig. 1:**
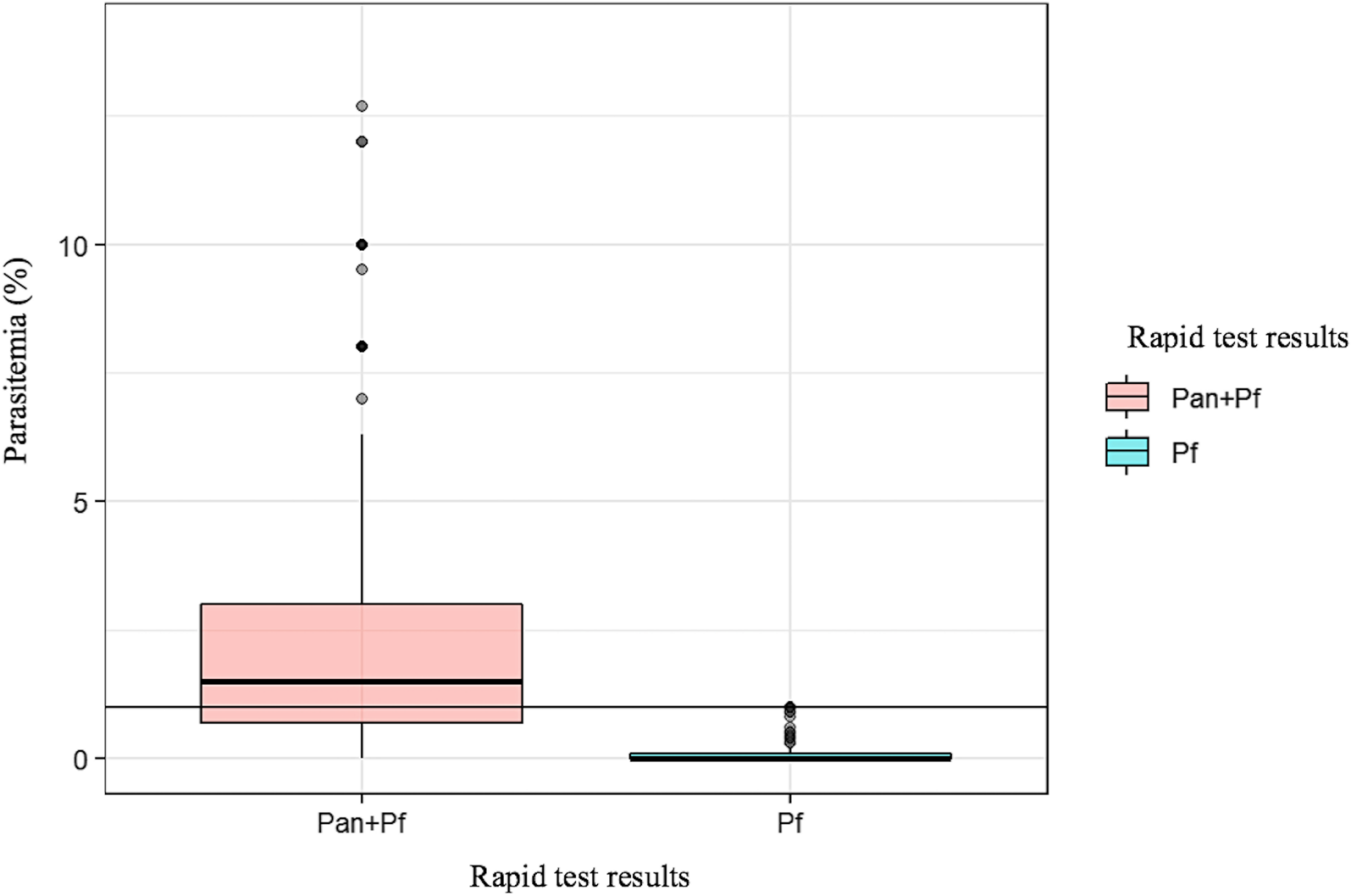
Parasitaemia distribution according to rapid diagnostic test results

Figure 1 shows parasitaemia distribution (%) according to the RDT results. Patients with Pan/Pf pattern had a parasitaemia range between 0 and 37% (mean 2.54%) while those with Pf pattern had ranges between 0–1% (mean 0.16%).

**Fig. 2:**
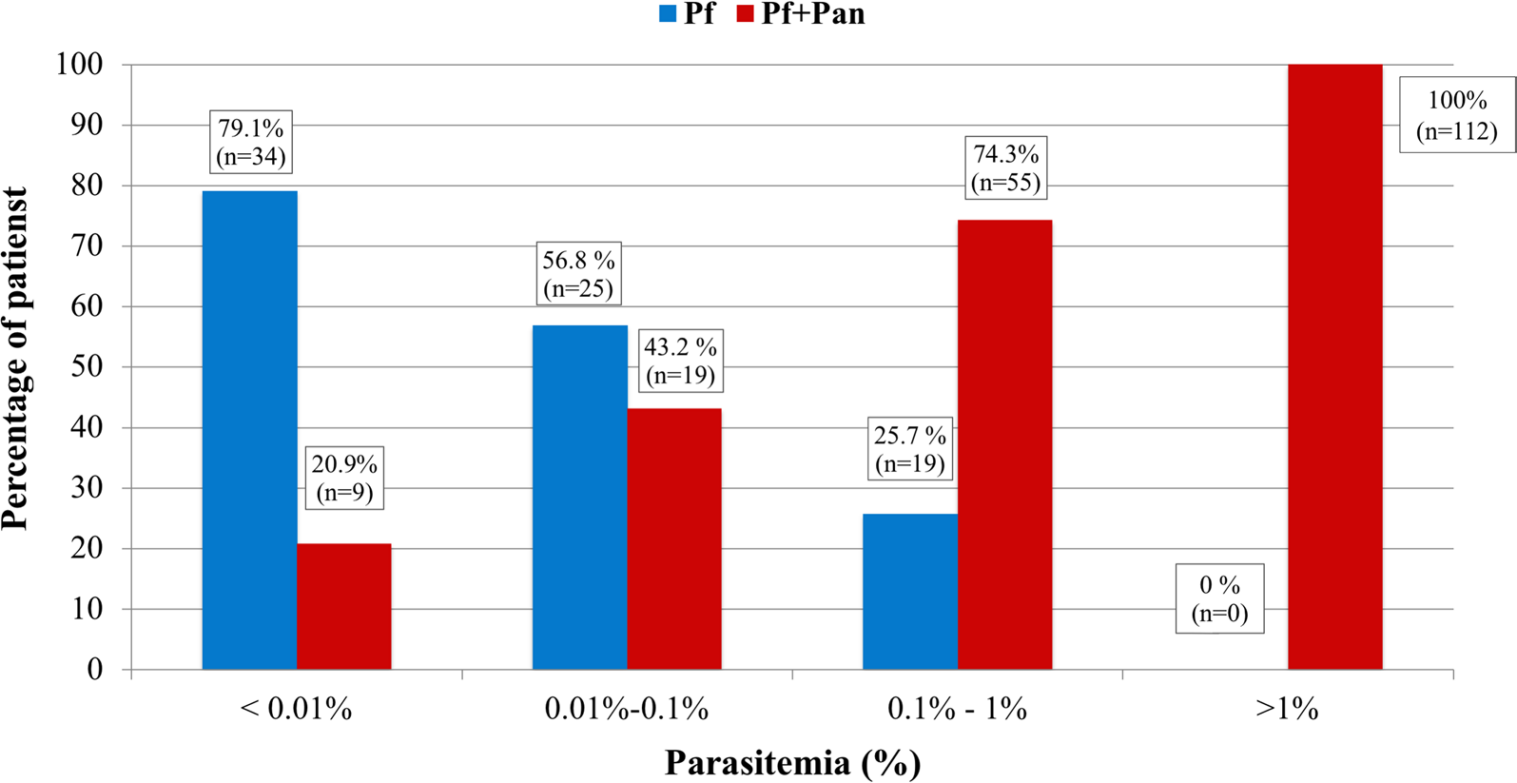
Distribution of rapid diagnostic test results by ranges of parasitaemia

The percentage of patients with a Pan/Pf pattern increased proportionally along with the increase in the parasitaemia range (Fig. 2). The ROC curve showed an AUC (area under the curve) value of 0.905 (95% CI: 0.87–0.94), and the most efficient parasitaemia cut-off point to discriminate between the two results of the RDT was 0.475% (Sen: 0.87; Spe: 0.80; PPV: 0.64; NPV: 0.94). As seen in Fig. 2, for parasitaemia ranges greater than 1%, not a single case of Pf pattern was found in the RDT. Therefore, the Pf pattern showed a negative predictive value (NPV) of 100% for high parasitaemia.

**Fig. 3:**
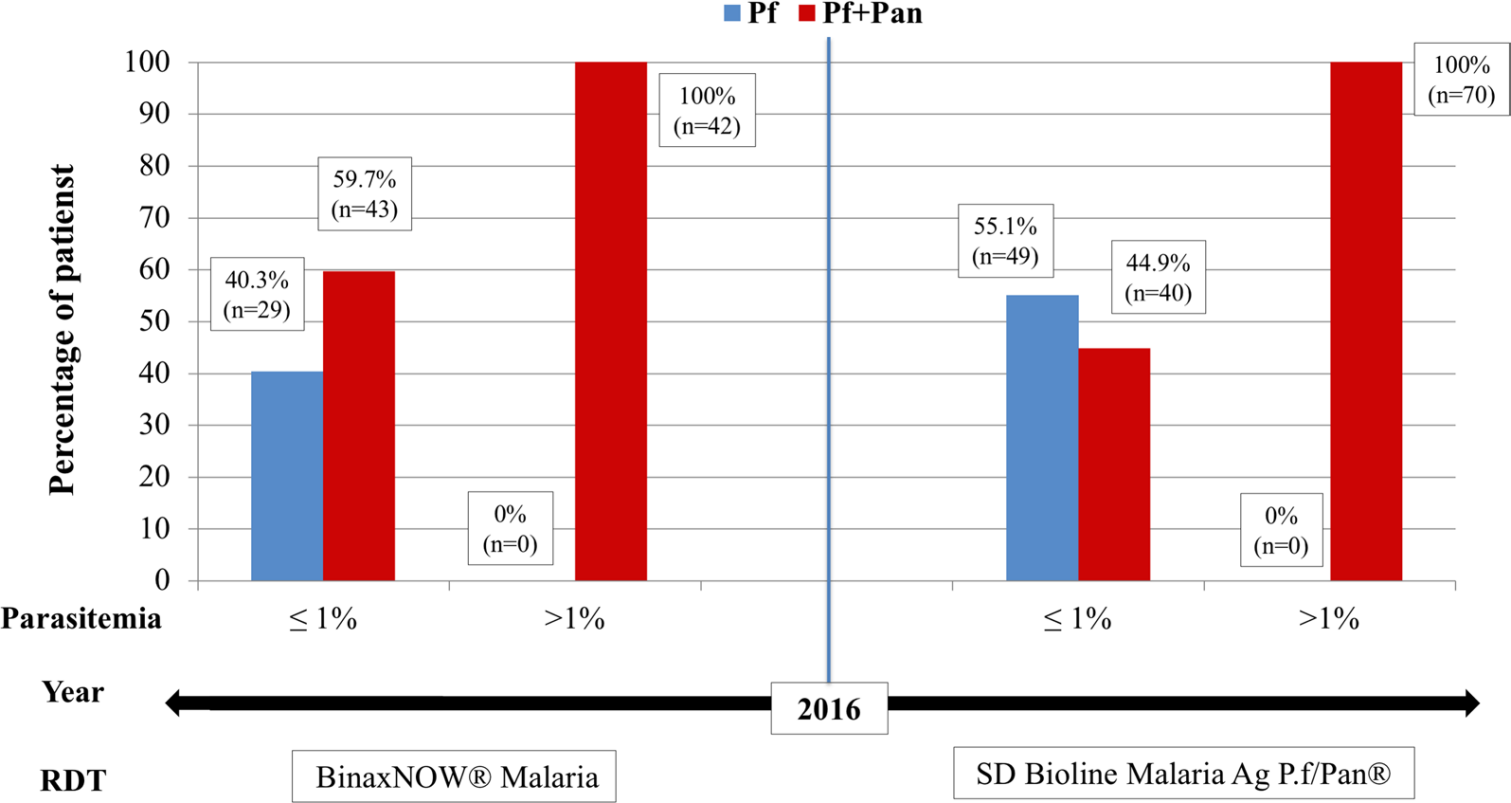
Temporal distribution of rapid diagnostic test results along the study period

The results of the RDT have been analysed separately in two temporal periods following the use of each of the test at the time: BinaxNOW® malaria test before 2016 and SD Bioline Malaria Ag P.f/Pan® from 2016 on). During the first period, 114 patients were included, 85 (74.6%) of them presenting a Pan/Pf pattern and 29 (25.4%) a Pf pattern. In the second period 159 patients were included, 110 (69.2%) presenting a Pan/Pf pattern and 49 (30.8%) a Pf pattern. Figure 3 shows the graphic distribution of the RDT results in both periods, categorized by two parasitaemia thresholds (< = 1% vs > 1%). In both temporal periods, and irrespective of the diagnostic test used, the described paradigm that patients with an isolated *P. falciparum* result never have parasitaemias > 1% is again observed.

### Correlation between the haematological parameters and parasitaemia

The correlation coefficients between each blood count variable and parasitaemia were calculated and are shown in Table 2. Some of these correlations resulted to be significants (p < 0.05). Lymphocytes, monocytes, eosinophils and platelets showed a negative correlation with parasitaemia, while neutrophils and the NLCR were positively correlated. Nevertheless, all the correlations resulted to be weak, with a maximum rho coefficient of -0.35 for lymphocytes and platelets, and of 0.40 for NLCR.

**Table 2:** Summary of haematological variables and their correlation to parasitaemia

	Rho*	p value **
Haemoglobin	0.03	0.62
Haematocrit	0.07	0.26
Leukocytes	− 0.02	0.74
Neutrophils	0.24	< 0.01
Lymphocytes	− 0.35	< 0.01
Monocytes	− 0.26	< 0.01
Eosinophils	− 0.26	< 0.01
Platelets	− 0.35	< 0.01
NLC ratio	0.40	< 0.01
MLC ratio	0.10	0.09

NLC: neutrophils-to-lymphocytes count; MLC: monocytes-to- lymphocytes count

*Spearman's rank correlation coefficient

^**^Significant at p‐value ≤ 0.05

### Predictive model for parasitaemia using the haematological parameters

A statistical model (generalized linear model) to predict the degree of parasitaemia was constructed, initially including haemoglobin, leukocytes, monocytes, eosinophils, platelets, and NLC and MLC ratios. Haematocrit, lymphocytes and neutrophils were eliminated because they behaved as covariates with a high degree of correlation. The results of fitting the generalized linear model to the variable representing the degree of parasitaemia are shown in Additional file 1: Table S1. The NLCR appeared as a risk factor for higher parasitaemia, finding that each increase by one unit in the NCLR was associated with a 19% increase in the degree of parasitaemia. Conversely, for MLCR and haemoglobin levels, parasitaemia decreased as they increased (OR 0.29 and 0.85, respectively).

**Fig. 4:**
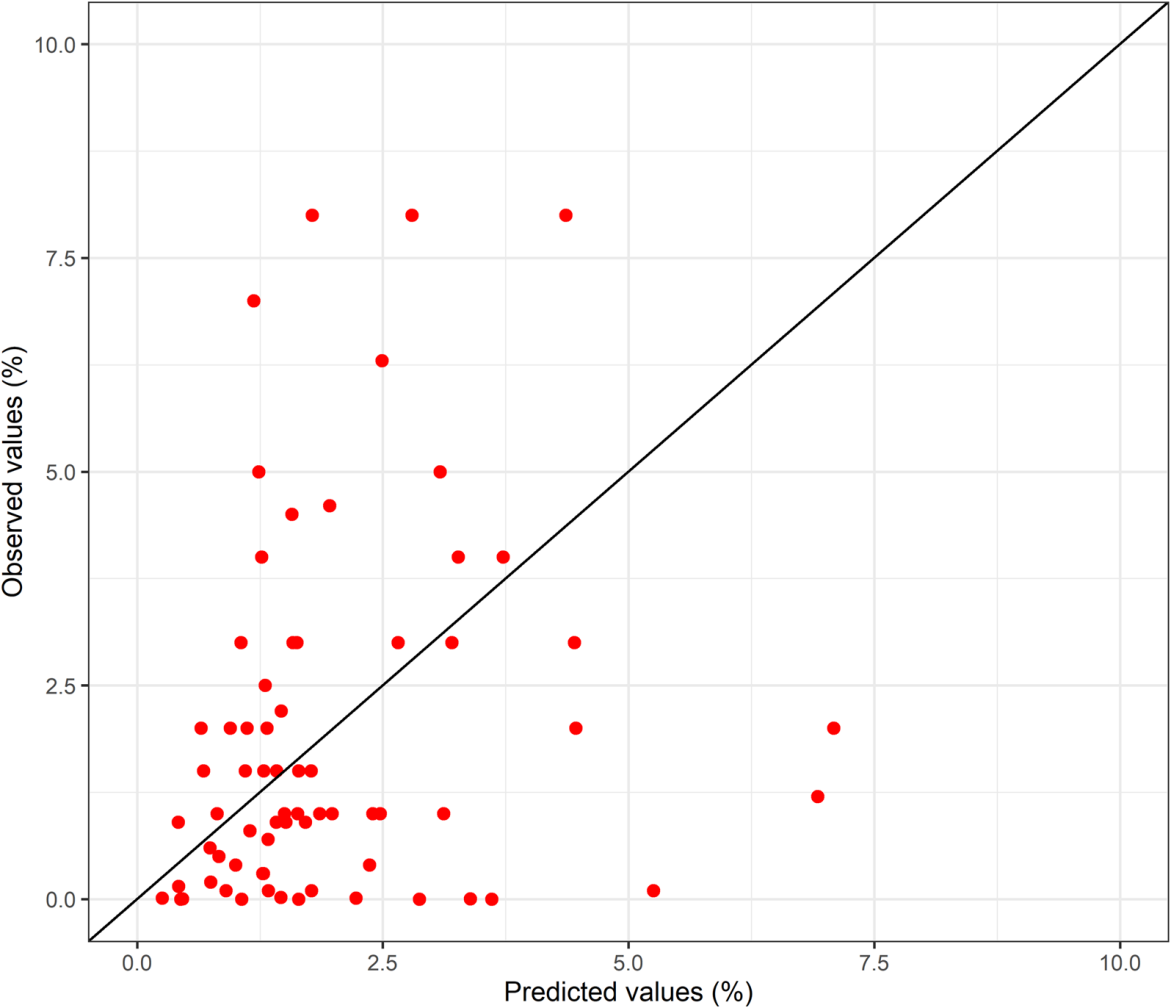
Real versus predicted data in the validation dataset

In Fig. 4, the plot graphic shows how the predicted data fits the real data. The RMSE for this model was 2.26%, with a range of possible values in the validation dataset from 0 to 8%, reflecting a poor predictive capacity.

### Relation between haematological location parameters and parasitaemia ranges

**Fig. 5:**
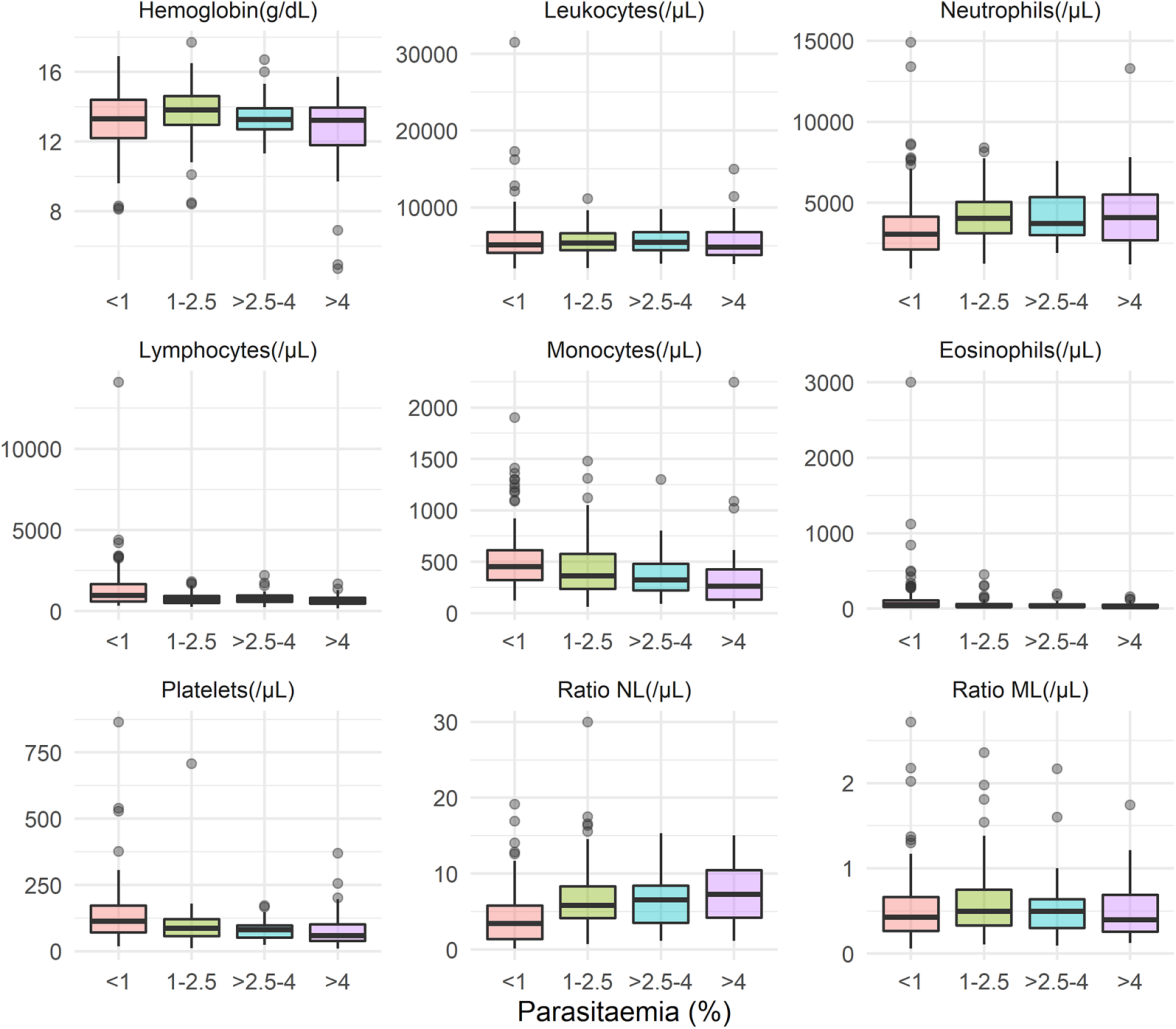
Blood count variables according to parasitaemia ranges

The relationship between blood count parameters and parasitaemia was again analysed, this time after parasitaemia was categorized in ranges as < 1%, 1%-2.5%, > 2.5%—4%, and > 4%. Figure 5 and Additional file 1: Table S2 show the medians and interquartile ranges of the different haematological variables for each of the parasitaemia ranges. Differences were observed according to the range of parasitaemia in the absolute values of neutrophils, lymphocytes, monocytes, eosinophils, platelets and NLCR. Patients with lower parasitaemias (< 1%) had significantly lower neutrophil counts and higher lymphocytes and monocytes than those in the 1–2.5% and > 4% ranges. Eosinophil counts only showed significant differences between the lowest and highest parasitaemia ranges (lower counts in the case of elevated parasitaemia). Both platelets and NLCR were significantly different (higher and lower, respectively) between patients with parasitaemia < 1% and all other ranges.

Given that the differences between the location parameters (i.e., the median) were generally between the < 1% range and the rest (1–2.5%, > 2.5–4% and > 4%), a predictive model was built to try to discriminate between parasitaemias < 1% and ≥ 1%. The coefficients of the statistical model are presented in Additional file 1: Table S3.

Eosinophils, platelets, and the NLC and MLC ratios were the only covariates selected for the model. The model was able to predict correctly 70% of the observations; the achieved sensitivity was 58%, while the specificity was 79%. Positive predictive value and negative predictive value were 69% and 70%, respectively.

## Discussion

In this cohort of patients with *P. falciparum* imported malaria, reactivity solely for HRP-2 on the combined RDT (Pf pattern) allowed discriminating those patients with parasitaemia ≤ 1%. On the contrary, the haematological parameters during the acute malaria episode did not show a correlation strong enough to indirectly estimate the level of parasitaemia.

Although microscopy continues to be the mainstay for malaria diagnosis, its widespread use is somewhat limited because of technical obstacles. The need for laboratory support in endemic regions and the scarce microbiological experience in non-endemic areas have promoted the search for alternative methods to assess *Plasmodium* parasitaemia. The demonstration that the quantification of plasma levels of PfHRP2 allowed estimating the total body parasite biomass in acute falciparum malaria [19, 20] was the starting point for works that aimed to establish a relation between RDT reactivity patterns and the degree of parasitaemia.

Richter et al. [9] had previously reported that co-reactivity of PfHPR-2/pAldolase in patients with febrile syndrome after returning from malaria endemic zones occurred predominantly in those with elevated parasitaemia (≥ 40,000 parasites/μl) due to *P. falciparum* infection (20/25; 80%). On the contrary, such reactivity pattern was less frequent in patients with mixed infections (2/4; 50%) or with mono-infections due to *P. falciparum* with parasitaemia < 40,000/μl (5/27; 18.5%). They concluded that co-reaction of PfHRP-2 and aldolase indicates the presence of a monoparasitic infection due to *P. falciparum* with high parasitaemia. Later on, Van Gool et al. [8] analysed the relationship between parasitaemia and RDT reactivity patterns (PfHPR-2/pAldolase) in 257 patients with *P. falciparum* imported malaria. As in our study, most patients acquired the infection in sub-Saharan Africa and belonged to the VFR traveller category. In this case, all patients with parasitaemia > 50,000/μl (> 1%) had PfHRP-2/pAldolase co-reactivity, while the absence of reactivity of pan-malarial antigen (HPR-2 reactivity pattern) allowed to exclude those patients with parasitaemia greater than 1% with a negative predictive value of 100%. This work reproduces the same results in a population of similar characteristics and also extends them to RDT that uses pLDH as pan-malarial antigen. Similarly, both studies contradict the hypothesis that PfHRP-2/pan-malarial antigen co-reactivity is associated with *P. falciparum* infections with high parasitaemia as this pattern also occurs in infections with very low parasitaemia. As shown in Fig. 2, up to 25.9% of patients with parasitaemia < 0.01% and up to 50% of patients with parasitaemia between 0.01%-0.1% showed reactivity against both antigens, again figures very similar to those of the study by Van Gool et al*.* [8]. Other studies using pLDH as panmalarial RDT antigen have reported comparable results [21].

It seems that the differences in antibody-binding avidity between PfHRP2 and pLDH may be reflected in the different sensitivity of the respective test bands. While blood concentrations ≥ 4 ng /mL of HRP-2 are needed to produce positive results in the specific *P. falciparum* band, concentrations 10 times higher (45 ng/mL) of pLDH are required for pan-malarial bands to be positive. This could be explained by the fact that PfHRP-2 possesses multiple epitopes capable of binding to immunoglobulins while pLDH possesses only one such epitope [21]. Based on the similar behavior between the two RDTs during the study a similar explanation can be assumed for aldolase. It should not be forgotten that reactivity of the PfHPR-2 band solely requires careful evaluation because it can happen in patients with active infections and low parasitaemia but also in individuals with a recent resolved infection, since antigenemia can persist for more than 4 weeks after parasitic clearance [22].

The *Plasmodium* parasite is a blood pathogen that causes different haematological manifestations due both to direct and indirect mechanisms. Target cells are not only red blood cells but white blood cells and platelets too. Anaemia, thrombocytopenia, lymphopenia, monocytosis, and eosinopenia are the most frequent haematological findings [14, 15, 23, 24]. Nevertheless, their occurrence can vary depending on several determinants such as nutritional status, demographic factors, the existence of haemoglobinopathies, the level of malaria endemicity, or the immune situation [25, 26]. The capacity of these haematological alterations to predict acute malaria in patients with acute febrile syndrome and epidemiological risk is widely documented, especially in the case of thrombocytopenia [27–31]. In contrast, studies that have attempted to estimate the severity of infection and parasitaemia have yielded inconsistent results.

Severe anaemia (Hb < 5 mg/dL) is the only haematological parameter that is part of the WHO criteria for severe malaria [6]. Malaria-induced anaemia can result from several factors, including the destruction of infected red blood cells as well as the rapid removal of both parasitized and non-parasitized red blood cells. Several previous studies demonstrate a significant increase in the prevalence of anaemia with the increase in parasite density [10, 32]. Most explore this relationship by categorizing parasitaemia with high parasitaemia ranges typically showing differences with the rest (low or moderate ranges). Even so, the works attempting to determine a linear relationship between parasitaemia and haemoglobin and/or haemotocrit show a weak negative correlation [33, 34]. In this study, no linear correlation nor significant difference between the different ranges of parasitaemia studied were found. This could be explained by the fact that patients with very high parasitaemia were poorly represented in our study (only 22.3% of patients in our study had a parasitaemia greater than 2.5%) as compared to other studies.

Despite not being a criterion for severe malaria, thrombocytopenia is one of the most consistent features of malaria. Destruction of platelet by macrophages, bone marrow alterations, antibody-mediated platelet destruction, oxidative stress, platelet aggregation, coagulation disturbances, and splenomegaly are all related to its pathogenesis. In this study, platelet count is significantly reduced in patients with high parasitaemia compared to those in the low and moderate parasitaemia ranges, findings that are similar to those previously described in other studies [10, 12]. Again, although a significant negative linear correlation with parasitemia is present, it was a weak one (rho − 0.35). Other studies that have studied the correlation between platelets and parasitaemia have shown comparable results.

Changes in leukocyte counts are often less pronounced than in the other blood cell lineages. In general, total leukocyte counts have been found to be low to normal in malaria [35]. Those studies that have analysed the association between white blood cell subsets and the severity of clinical presentation or parasitaemia show conflicting results. Some studies report associations between high circulating monocyte, lymphocyte, neutrophil or eosinophil counts and high parasitaemia or severe clinical conditions, but others report significantly lower cell counts in patients with high parasitaemia or complicated malaria [10–16, 36]. This disagreement might be due to differences in the haematological profile of circulating cells between sex or geographical areas [37]. Regardless of the number of malaria episodes experienced, age and season also affect white blood cell subsets [38]. In this study, a statistically significant trend to lymphopenia, monocytopenia, eosinopenia, and neutrophilia in patients within the higher range of parasitemia were found. The existing linear relationships show, however, weak correlations (rho ranges between -0.35 and 0.24), similar to those described in the literature.

In recent years, multiple studies have been published on the diagnostic and prognostic value of different haematological ratios and their ability to provide added information to the one given by standard blood count values. The ratio neutrophil-to-lymphocyte count (NLCR) has been proposed as marker of severe systemic inflammation in critical patients admitted to intensive care units [39], as a better predictor of bacteremia in patients treated in emergency services [40], or as a prognostic marker in patients with community acquired pneumonia [41]. Increased NLCR also behaves as an independent marker of survival in non-infectious oncological [42] or cardiovascular [43] conditions. Its usefulness in malaria episodes has also been evaluated although with less robust results. In their study of 440 patients with imported malaria, Wolfswinken et al. [29] found a positive but very weak correlation with parasitaemia (rho 0.165) and its prognostic ability to predict adverse events determined by AUC was worse than that of C-reactive protein (0.57 vs 0.84). In this study, the correlation between NLCR and parasitaemia is higher (rho 0.4) and there is a clear trend towards a progressive increase in the different ranges of parasitaemia explored. In addition, in the predictive model, the NLCR behaves as a risk factor for greater parasitaemia. These different results could be explained by the absence of parasitaemia-lymphocyte and parasitaemia-neutrophil correlation in Wolfswinken’s study while such correlations, although weak, do exist in this work.

Monocytes are essential components of the innate immune response and act as a link to the adaptive response by means of antigenic presentation to lymphocytes and the production of cytokines. However, they can also participate in the pathogenesis of severe conditions [44]. The monocyte-to-lymphocyte count ratio (MLCR) began to be investigated in patients with malaria in the works of Warime et al*.* [45, 46]. They found that, during the follow-up of children with asymptomatic *P. falciparum* infection, the presence of a high MLC ratio correlated with a greater susceptibility to clinical malaria and a lower clinical effectiveness of the RTS,S malaria vaccine. These results posited the MLCR as a possible marker of the individual's ability to mount an effective immune response against malaria. In their work, Antwi-Baffour et al*.* [13] examined the monocyte to lymphocyte ratio during *P. falciparum* infections in children up to 5 years compared to healthy controls. There was significant difference between case and controls and a significant positive correlation with parasitaemia, although very weak (rho 0.20). This work does not replicate these findings. In this study, no significant linear correlation (p 0.09) or differences between the different groups after categorizing parasitemia were found. Moreover, in the constructed predictive model, the MLC ratio behaves as a protective factor regarding parasitic load. These discordant findings could be explained by the differences in the populations included in both studies (children in malaria-endemic area versus VFR adults with imported malaria) and in their immune status with respect to *Plasmodium* infection. The findings of Berens-Riha et al. [26] are in line with the above. Their study, which included 210 adult travellers with imported malaria, found no linear correlation between MLCR and parasitaemia, and semi-immune individuals had significantly lower values than non-immune individuals.

The current study presents the methodological limitations inherent to its retrospective nature, although in this case it has probably little influence on the results. The majority of patients included in the study are sub-Saharan VFR travellers and therefore, the findings may not be simply extrapolated to other population groups. On the other hand, the large number of patients included in the study strengthens the results. Despite numerous studies describing the behaviour of haemotologic parameters in patients with malaria, there are few studies describing their association with parasitaemia. Similarly, as far as we know, there are no other studies attempting to estimate such association using multivariate predictive models including different variables and haematological ratios. This research extends the semi-quantitative approach to parasitaemia based on the pattern of the results of RDT to those HPR2-pLDH tests since, to date, similar evidence had only been demonstrated with tests that use pAldolase as the pan-malarial band. HRP2-based diagnostic tests can remain positive for an extended period of time following successful *P. falciparum* parasite clearance because the antigen lingers weeks after resolution of infection. This situation is particularly relevant in high-transmission setting were antigen presence does not necessarily imply active parasite infection. As a result, is not recommended to extrapolate findings from this work to other contexts than that of imported malaria [47]. Finally, the reported results may have clinical utility facilitating clinical decision making in patients with imported malaria.

## Conclusions

The results of this study show that the reactivity pattern of BinaxNOW® malaria test (HPR-2/aldolase) and SD Bioline Malaria Ag P.f/Pan (HPR-2/pLDH) allows a rapid semi-quantitative assessment of *P. falciparum* parasitaemia in travellers with imported malaria, particularly to point-out patients with lower parasite loads. The presence of HRP-2 reactivity in the absence of LDH or aldolase was a reliable predictive marker to detect parasitaemias < 1% with a 100% PPV. On the contrary, neither haematological parameters, nor NLC or MLC ratios, nor the multivariate predictive models developed, were able to estimate parasitaemia with sufficient precision.

This findings may be relevant and useful when treating patients suspected of having imported malaria in the acute care setting, especially in those centres where microscopic diagnosis is not readily available or where expertise in managing malaria is lacking. Further studies involving a larger number of patients and different populations are needed to confirm that these data are extrapolable to different scenarios.

### Supplementary Information

**Additional file 1:** Details of correlation between the haematological parameters and parasitaemia. **Table S1.** Parameter estimates of the definitive generalized linear model for the variable “parasitemia”. **Table S2.** Hematological parameters by parasitemia ranges. **Table S3.** Parameter estimates of the predictive model for the parasitaemia variable (< 1% and ≥ 1%). 

## Data Availability

The clinical dataset will be made available upon reasonable request to the corresponding authors.

## Abbreviations

RDT: Rapid diagnostic tests
PCR: Polymerase chain reaction
VFR: Visiting friends and relatives
pLDH: Plasmodial lactate dehydrogenase
HRP-2: Histidine-rich protein 2
TMU: Tropical Medicine Unit
MLCR: Monocytes-to- lymphocytes count ratio
NLCR: Neutrophils-to-lymphocytes count ratio
ROC: Receiver operating characteristic
RMSE: Root mean square error
AIC: Akaike’s information criterion
ICU: Intensive Care Unit
AUC: Area under the curve
OR: Odds ratio
CI: Confidence interval
