# Supplementary material for: Estimation of parasitaemia in imported falciparum malaria using the results of a combined rapid diagnostic test. No big help from haematological parameters
Source: Malar J. 2023 Nov 16;22:351. doi: 10.1186/s12936-023-04781-2 (PMC10655380; doi:10.1186/s12936-023-04781-2)
Supplement: Supplementary file 1 — Additional file 1: Details of correlation between the haematological parameters and parasitaemia. Table S1. Parameter estimates of the definitive generalized linear model for the variable “parasitemia”. Table S2. Hematological parameters by parasitemia ranges. Table S3. Parameter estimates of the predictive model for the parasitaemia variable (< 1% and ≥ 1%). [file 12936_2023_4781_MOESM1_ESM.docx]

**Table S1. Parameter estimates of the definitive generalized linear model for the variable “parasitemia”.**

|  | **Estimate** | **Standard Error** | **t value** | **OR** | **CI (95%)** | **p value** |
| --- | --- | --- | --- | --- | --- | --- |
| **(Intercept)** | -2.45 | 0.61 | -4.00 | 0.087 | 0.025 - 0.271 | <0.001 |
| **Hemoglobin** | -0.15 | 0.05 | -3.31 | 0.857 | 0.786 - 0.943 | 0.001 |
| **NLC ratio** | 0.18 | 0.02 | 7.15 | 1.192 | 1.136 - 1.250 | <0.001 |
| **MLC ratio** | -1.21 | 0.31 | -3.91 | 0.297 | 0.158 - 0.535 | <0.001 |

NLC: neutrophils-to- lymphocytes count; MLC: monocytes-to- lymphocytes count; OR: odds ratio;

CI: confidence interval.

**Table S2. Hematological parameters by parasitemia ranges.**

|  | **<1% (n=133)** | **1-2.5% (n=79)** | **>2.5-4%**  **(n=26)** | **>4%**  **(n=35)** | **p value *** |
| --- | --- | --- | --- | --- | --- |
| **Hemoglobin**  · Median  · Q1-Q3 | 13.30  12.20-14.40 | 13.80  12.95-14.6 | 13.25  12.70-13.90 | 13.20  11.80-13.95 | 0.057 |
| **Hematocrit**  · Median  · Q1-Q3 | 39.20  36.00-42.00 | 40.10  37.40-43.45 | 39.05  36.80-41.00 | 39.80  35.75-41.45 | 0.127 |
| **Leukocytes**  · Median  · Q1-Q3 | 5090  4070-6740 | 5300  4395-6630 | 5440  4397-6745 | 4770  3765-6780 | 0.821 |
| **Neutrophils**  · Median  · Q1-Q3 | 3041  2120-4140 | 4020  3130-5058 | 3720  3010-5355 | 4072  2695-5515 | <0.001 |
| **Lymphocytes**  · Median  · Q1-Q3 | 965  610-1660 | 730  505-934 | 770  575-972 | 620  460-825 | <0.001 |
| **Monocytes**  · Median  · Q1-Q3 | 450  320-610 | 360  235-575 | 320  222-480 | 260  130-425 | <0.001 |
| **Eosinophils**  · Median  · Q1-Q3 | 50  20-110 | 30  20-60 | 30  20-57 | 20  10-50 | 0.013 |
| **Platelets**  · Median  · Q1-Q3 | 113,000  71,000-172,000 | 86,000  57,500-120,500 | 81,000  51,250-97,000 | 59,000  38,500-102,000 | <0.001 |
| **NLC ratio**  · Median  · Q1-Q3 | 3.38  1.39-5.79 | 5.81  4.13-8.31 | 6.52  3.49-8.40 | 7.24  4.16-10.45 | <0.001 |
| **MLC ratio**  · Median  · Q1-Q3 | 0.424  0.263-0.661 | 0.493  0.330-0.751 | 0.496  0.297-0.637 | 0.395  0.255-0.690 | 0.304 |

NLC: neutrophils-to- lymphocytes count; MLC: monocytes-to- lymphocytes count.

* p-value by Kruskal Wallis Test.

**Table S3. Parameter estimates of the predictive model for the parasitaemia variable (<1% and ≥ 1%).**

|  | **Estimate** | **Standard Error** | **z value** | **OR** | **CI (95%)** | **p value** |
| --- | --- | --- | --- | --- | --- | --- |
| **(Intercept)** | -0.078 | 0.381 | -0.205 | 0.93 | 0.44 – 1.95 | 0.838 |
| **Eosinophils** | -0.002 | 0.002 | -1.106 | 1.00 | 0.99 – 1.00 | 0.269 |
| **Platelets** | -0.004 | 0.002 | -1.930 | 1.00 | 0.99 – 1.00 | 0.054 |
| **NLC ratio** | 0.211 | 0.053 | 3.971 | 1.23 | 1.11 – 1.37 | <0.001 |
| **MLC ratio** | -0.766 | 0.479 | -1.599 | 0.46 | 0.18 – 1.19 | 0.110 |

NLC: neutrophils-to- lymphocytes count; MLC: monocytes-to- lymphocytes count; OR: odds ratio;

CI: confidence interval.
